# Supplementary material for: KStable: A Computational Method for Predicting Protein Thermal Stability Changes by K-Star with Regular-mRMR Feature Selection
Source: Entropy (Basel). 2018 Dec 19;20(12):988. doi: 10.3390/e20120988 (PMC7512587; doi:10.3390/e20120988)
Supplement: Supplementary file 1 [file entropy-20-00988-s001.pdf]

**Table S1.** The selected features of 2-mRMR by mRMR for five tests.

| | PT 1 | PT 2 | PT 3 | PT 4 | PT 5 | | PT 1 | PT 2 | PT 3 | PT 4 | PT 5 |
|---|---|---|---|---|---|---|---|---|---|---|---|
| **1** | v645 | v645 | v645 | v645 | v647 | **16** | v3 | v518 | v505 | v702 | v157 |
| **2** | v650 | v650 | v302 | v302 | v302 | **17** | v390 | v453 | v650 | v578 | v530 |
| **3** | v42 | v42 | v168 | v650 | v42 | **18** | v525 | v531 | v626 | v79 | v707 |
| **4** | v168 | v302 | v42 | v168 | v650 | **19** | v97 | v168 | v531 | v525 | v465 |
| **5** | v651 | v608 | v574 | v42 | v518 | **20** | v587 | v707 | v108 | v600 | v52 |
| **6** | v117 | v130 | v239 | v52 | v547 | **21** | v331 | v530 | v117 | v265 | v523 |
| **7** | v109 | v52 | v518 | v297 | v274 | **22** | v5 | v3 | v219 | v17 | v319 |
| **8** | v731 | v195 | v453 | v574 | v651 | **23** | v203 | v331 | v547 | v566 | v732 |
| **9** | v566 | v390 | v731 | v575 | v453 | **24** | v280 | v686 | v195 | v565 | v109 |
| **10** | v547 | v651 | v224 | v522 | v224 | **25** | v139 | v687 | v503 | v432 | v430 |
| **11** | v453 | v106 | v651 | v41 | v97 | **26** | v81 | v239 | v390 | v99 | v3 |
| **12** | v321 | v117 | v75 | v651 | v725 | **27** | v239 | v157 | v41 | v230 | v575 |
| **13** | v319 | v72 | v319 | v203 | v566 | **28** | v579 | v297 | v52 | v75 | v578 |
| **14** | v75 | v41 | v530 | v530 | v41 | **29** | v465 | v102 | v331 | v195 | v439 |
| **15** | v52 | v299 | v707 | v453 | v531 | **30** | v687 | v357 | v97 | v704 | v131 |

**Table S2.** The selected features of diff-mRMR by mRMR for five tests.

| | PT 1 | PT 2 | PT 3 | PT 4 | PT 5 | | PT 1 | PT 2 | PT 3 | PT 4 | PT 5 |
|---|---|---|---|---|---|---|---|---|---|---|---|
| **1** | M324 | M324 | M324 | M324 | M324 | **16** | M163 | M323 | M115 | M2 | M115 |
| **2** | M258 | M258 | M258 | M258 | M258 | **17** | M194 | M194 | M133 | M122 | M160 |
| **3** | M191 | M166 | M75 | M35 | M75 | **18** | M322 | M160 | M282 | M320 | M320 |
| **4** | M166 | M191 | M166 | M48 | M191 | **19** | M321 | M322 | M225 | M171 | M165 |
| **5** | M76 | M165 | M48 | M49 | M48 | **20** | M35 | M100 | M120 | M321 | M35 |
| **6** | M48 | M133 | M194 | M344 | M198 | **21** | M13 | M163 | M171 | M322 | M163 |
| **7** | M183 | M48 | M110 | M115 | M166 | **22** | M49 | M96 | M320 | M183 | M321 |
| **8** | M165 | M98 | M192 | M194 | M49 | **23** | M225 | M183 | M100 | M323 | M98 |
| **9** | M78 | M172 | M163 | M92 | M92 | **24** | M323 | M171 | M253 | M225 | M2 |
| **10** | M122 | M195 | M49 | M365 | M183 | **25** | M133 | M40 | M108 | M16 | M100 |
| **11** | M96 | M344 | M2 | M163 | M194 | **26** | M108 | M13 | M98 | M195 | M282 |
| **12** | M160 | M192 | M36 | M96 | M298 | **27** | M250 | M320 | M195 | M192 | M212 |
| **13** | M98 | M49 | M38 | M161 | M171 | **28** | M361 | M32 | M323 | M361 | M96 |
| **14** | M320 | M274 | M122 | M290 | M38 | **29** | M345 | M34 | M183 | M250 | M120 |
| **15** | M192 | M149 | M274 | M100 | M121 | **30** | M339 | M253 | M165 | M110 | M192 |

**Table S3.** The 30 highest-ranking mRMR features selected by regular-mRMR feature selection for the three feature types of 2-mRMR, diff-mRMR, and all-mRMR.

| | Diff-mRMR | 2-mRMR | All-mRMR | | Diff-mRMR | 2-mRMR | All-mRMR |
|---|---|---|---|---|---|---|---|
| **1** | M324 | v645 | M324 | **16** | M163 | v3 | M149 |
| **2** | M258 | v650 | v651 | **17** | M194 | v390 | M250 |
| **3** | M191 | v42 | M96 | **18** | M322 | v525 | M40 |
| **4** | M166 | v168 | M320 | **19** | M321 | v97 | M100 |
| **5** | M76 | v651 | M194 | **20** | M35 | v587 | M253 |
| **6** | M48 | v117 | M323 | **21** | M13 | v331 | M195 |
| **7** | M183 | v109 | M171 | **22** | M49 | v5 | M339 |
| **8** | M165 | v731 | M321 | **23** | M225 | v203 | M120 |
| **9** | M78 | v566 | v453 | **24** | M323 | v280 | M110 |
| **10** | M122 | v547 | M225 | **25** | M133 | v139 | M108 |
| **11** | M96 | v453 | M2 | **26** | M108 | v81 | v117 |
| **12** | M160 | v321 | M322 | **27** | M250 | v239 | v157 |
| **13** | M98 | v319 | M13 | **28** | M361 | v579 | M16 |
| **14** | M320 | v75 | M361 | **29** | M345 | v465 | M345 |
| **15** | M192 | v52 | M49 | **30** | M339 | v687 | M57 |

**Table S4.** The AAIndex coding for diff-mRMR and 2-mRMR.

| AAindex feature | diff-mRMR | 2-mRMR wild-type) | 2-mRMR (mutant) |
|---|---|---|---|
| alpha-CH chemical shifts | M1 | V1 | V2 |
| Hydrophobicity index | M2 | V3 | V4 |
| Membrane-buried preference parameters | M3 | V5 | V6 |
| Conformational parameter of the inner helix | M4 | V7 | V8 |
| Conformational parameter of the beta-structure | M5 | V9 | V10 |
| Conformational parameter of the beta-turn | M6 | V11 | V12 |
| Average flexibility indices | M7 | V13 | V14 |
| Membrane-buried preference parameters (Argos et al. 1982) | M8 | V15 | V16 |
| Conformational parameter of the inner helix (Beghin and Dirkx 1975) | M9 | V17 | V18 |
| Conformational parameter of the beta-structure (Beghin and Dirkx 1975) | M10 | V19 | V20 |
| Conformational parameter of the beta-turn (Beghin and Dirkx 1975) | M11 | V21 | V22 |
| Average flexibility indices (Bhaskaran and Ponnuswamy 1988) | M12 | V23 | V24 |
| Information value for accessibility average fraction 35 (Biou et al. 1988) | M13 | V25 | V26 |
| Retention coefficient in TFA (Browne et al. 1982) | M14 | V27 | V28 |
| Retention coefficient in HFBA (Browne et al. 1982) | M15 | V29 | V30 |
| Transfer free energy to surface (Bull and Breese 1974) | M16 | V31 | V32 |
| Apparent partial specific volume (Bull and Breese 1974) | M17 | V33 | V34 |
| alpha-NH chemical shifts (Bundi and Wuthrich 1979) | M18 | V35 | V36 |
| Spin–spin coupling constants 3JHalpha-NH (Bundi and Wuthrich 1979) | M19 | V37 | V38 |
| Normalized frequency of the extended structure (Burgess et al. 1974) | M20 | V39 | V40 |
| Steric parameter (Charton 1981) | M21 | V41 | V42 |
| Polarizability parameter (Charton and Charton 1982) | M22 | V43 | V44 |
| The Chou–Fasman parameter of the coil conformation (Charton and Charton 1983) | M23 | V45 | V46 |
| A parameter defined from the residuals obtained from the best correlation of the Chou–Fasman parameter of the beta-sheet (Charton and Charton 1983) 2 | M24 | V47 | V48 |
| The number of atoms in the side chain labelled 1+1 (Charton and Charton 1983) | M25 | V49 | V50 |
| The number of atoms in the side chain labelled 2+1 (Charton and Charton 1983) | M26 | V51 | V52 |
| The number of atoms in the side chain labelled 3+1 (Charton and Charton 1983) | M27 | V53 | V54 |
| The number of bonds in the longest chain (Charton and Charton 1983) | M28 | V55 | V56 |
| A parameter of charge transfer donor capability (Charton and Charton 1983) | M29 | V57 | V58 |
| Average volume of buried residue (Chothia 1975) | M30 | V59 | V60 |
| Residue-accessible surface area in the folded protein (Chothia 1976) | M31 | V61 | V62 |
| Normalized frequency of the beta-turn (Chou and Fasman 1978a) | M32 | V63 | V64 |

| | | | |
|---|---|---|---|
| Normalized frequency of the alpha-helix (Chou and Fasman 1978b) | M33 | V65 | V66 |
| Normalized frequency of the beta-sheet (Chou and Fasman 1978b) | M34 | V67 | V68 |
| Normalized frequency of the beta-turn (Chou and Fasman 1978b) | M35 | V69 | V70 |
| Normalized frequency of the N-terminal helix (Chou and Fasman 1978b) | M36 | V71 | V72 |
| Normalized frequency of the C-terminal helix (Chou and Fasman 1978b) | M37 | V73 | V74 |
| Normalized frequency of the N-terminal non-helical region (Chou and Fasman 1978b) | M38 | V75 | V76 |
| Normalized frequency of the C-terminal non-helical region (Chou and Fasman 1978b) | M39 | V77 | V78 |
| Normalized frequency of the C-terminal beta-sheet (Chou and Fasman 1978b) | M40 | V79 | V80 |
| Normalized frequency of the N-terminal non-beta region (Chou and Fasman 1978b) | M41 | V81 | V82 |
| Frequency of the 2nd residue in turn (Chou and Fasman 1978b) | M42 | V83 | V84 |
| Frequency of the 4th residue in turn (Chou and Fasman 1978b) | M43 | V85 | V86 |
| Normalized frequency of the 2nd and 3rd residues in turn (Chou and Fasman 1978b) | M44 | V87 | V88 |
| Normalized hydrophobicity scales for alpha-proteins (Cid et al. 1992) | M45 | V89 | V90 |
| Normalized hydrophobicity scales for alpha+beta-proteins (Cid et al. 1992) | M46 | V91 | V92 |
| Normalized average hydrophobicity scales (Cid et al. 1992) | M47 | V93 | V94 |
| Normalized frequency of the middle helix (Crawford et al. 1973) | M48 | V95 | V96 |
| Normalized frequency of the beta-sheet (Crawford et al. 1973) | M49 | V97 | V98 |
| Normalized frequency of the turn (Crawford et al. 1973) | M50 | V99 | V100 |
| Amino acid composition (Dayhoff et al. 1978a) | M51 | V101 | V102 |
| Membrane preference for cytochrome b: MPH89 (Degli Esposti et al. 1990) | M52 | V103 | V104 |
| Average membrane preference: AMP07 (Degli Esposti et al. 1990) | M53 | V105 | V106 |
| Solvation free energy (Eisenberg and McLachlan 1986) | M54 | V107 | V108 |
| Direction of hydrophobic moment (Eisenberg and McLachlan 1986) | M55 | V109 | V110 |
| Molecular weight (Fasman 1976) | M56 | V111 | V112 |
| Melting point (Fasman 1976) | M57 | V113 | V114 |
| Optical rotation (Fasman 1976) | M58 | V115 | V116 |
| Hydrophobic parameter pi (Fauchere and Pliska 1983) | M59 | V117 | V118 |
| Smoothed upsilon steric parameter (Fauchere et al. 1988) | M60 | V119 | V120 |
| Normalized van der Waals volume (Fauchere et al. 1988) | M61 | V121 | V122 |
| STERIMOL maximum width of the side chain (Fauchere et al. 1988) | M62 | V123 | V124 |
| Number of hydrogen bond donors (Fauchere et al. 1988) | M63 | V125 | V126 |
| Positive charge (Fauchere et al. 1988) | M64 | V127 | V128 |
| Negative charge (Fauchere et al. 1988) | M65 | V129 | V130 |
| pK-a(RCOOH) (Fauchere et al. 1988) | M66 | V131 | V132 |
| Helix-coil equilibrium constant (Finkelstein and Ptitsyn 1977) | M67 | V133 | V134 |
| Helix initiation parameter at position i i+1 i+2 (Finkelstein et al. 1991) | M68 | V135 | V136 |
| Helix termination parameter at position j-2 j-1 j (Finkelstein et al. 1991) | M69 | V137 | V138 |
| Partition coefficient (Garel et al. 1973) | M70 | V139 | V140 |

| | | | |
|---|---|---|---|
| Alpha-helix indices (Geisow and Roberts 1980) | M71 | V141 | V142 |
| Alpha-helix indices for alpha-proteins (Geisow and Roberts 1980) | M72 | V143 | V144 |
| Alpha-helix indices for beta-proteins (Geisow and Roberts 1980) | M73 | V145 | V146 |
| Beta-strand indices (Geisow and Roberts 1980) | M74 | V147 | V148 |
| Beta-strand indices for beta-proteins (Geisow and Roberts 1980) | M75 | V149 | V150 |
| Aperiodic indices (Geisow and Roberts 1980) | M76 | V151 | V152 |
| Aperiodic indices for alpha-proteins (Geisow and Roberts 1980) | M77 | V153 | V154 |
| Aperiodic indices for beta-proteins (Geisow and Roberts 1980) | M78 | V155 | V156 |
| Composition (Grantham 1974) | M79 | V157 | V158 |
| Polarity (Grantham 1974) | M80 | V159 | V160 |
| Partition energy (Guy 1985) | M81 | V161 | V162 |
| Heat capacity (Hutchens 1970) | M82 | V163 | V164 |
| Normalized relative frequency of the extended structure (Isogai et al. 1980) | M83 | V165 | V166 |
| Normalized relative frequency of the bend R (Isogai et al. 1980) | M84 | V167 | V168 |
| Normalized relative frequency of the double bend (Isogai et al. 1980) | M85 | V169 | V170 |
| Normalized relative frequency of the coil (Isogai et al. 1980) | M86 | V171 | V172 |
| Average accessible surface area (Janin et al. 1978) | M87 | V173 | V174 |
| Percentage of buried residues (Janin et al. 1978) | M88 | V175 | V176 |
| Percentage of exposed residues (Janin et al. 1978) | M89 | V177 | V178 |
| Ratio of buried and accessible molar fractions (Janin 1979) | M90 | V179 | V180 |
| Transfer free energy (Janin 1979) | M91 | V181 | V182 |
| pK (-COOH) (Jones 1975) | M92 | V183 | V184 |
| Relative frequency of occurrence (Jones et al. 1992) | M93 | V185 | V186 |
| Relative mutability (Jones et al 1992) | M94 | V187 | V188 |
| Amino acid distribution (Jukes et al. 1975) | M95 | V189 | V190 |
| Sequence frequency (Jungck 1978) | M96 | V191 | V192 |
| Average relative probability of a helix (Kanehisa and Tsong 1980) | M97 | V193 | V194 |
| Average relative probability of a beta-sheet (Kanehisa and Tsong 1980) | M98 | V195 | V196 |
| Average relative probability of an inner helix (Kanehisa and Tsong 1980) | M99 | V197 | V198 |
| Average relative probability of an inner beta-sheet (Kanehisa and Tsong 1980) | M100 | V199 | V200 |
| Flexibility parameter for no rigid neighbors (Karplus and Schulz 1985) | M101 | V201 | V202 |
| Flexibility parameter for one rigid neighbor (Karplus and Schulz 1985) | M102 | V203 | V204 |
| Flexibility parameter for two rigid neighbors (Karplus and Schulz 1985) | M103 | V205 | V206 |
| The Kerr-constant increments (Khanarian and Moore 1980) | M104 | V207 | V208 |
| Net charge (Klein et al 1984) | M105 | V209 | V210 |
| Side chain interaction parameter (Krigbaum and Rubin 1971) | M106 | V211 | V212 |
| Side chain interaction parameter (Krigbaum and Komoriya 1979) | M107 | V213 | V214 |
| Side chain volume (Krigbaum and Komoriya 1979) | M108 | V215 | V216 |

| | | | |
|---|---|---|---|
| Hydropathy index (Kyte and Doolittle 1982) | M109 | V217 | V218 |
| Transfer free energy CHP/water (Lawson et al. 1984) | M110 | V219 | V220 |
| Hydrophobic parameter (Levitt 1976) | M111 | V221 | V222 |
| Distance between C-alpha and the centroid of the side chain (Levitt 1976) | M112 | V223 | V224 |
| Side chain angle theta(AAR) (Levitt 1976) | M113 | V225 | V226 |
| Side chain torsion angle phi(AAAR) (Levitt 1976) | M114 | V227 | V228 |
| van der Waals parameter R0 (Levitt 1976) | M115 | V229 | V230 |
| Normalized frequency of the beta-sheet with weights (Levitt 1978) | M116 | V231 | V232 |
| Normalized frequency of the beta-sheet unweighted (Levitt 1978) | M117 | V233 | V234 |
| Normalized frequency of the reverse turn unweighted (Levitt 1978) | M118 | V235 | V236 |
| Frequency of occurrence in beta-bends (Lewis et al. 1971) | M119 | V237 | V238 |
| Conformational preference for all beta-strands (Lifson and Sander 1979) | M120 | V239 | V240 |
| Conformational preference for antiparallel beta-strands (Lifson and Sander 1979) | M121 | V241 | V242 |
| Normalized frequency of the alpha-helix (Maxfield and Scheraga 1976) | M122 | V243 | V244 |
| Normalized frequency of the left-handed alpha-helix (Maxfield and Scheraga 1976) | M123 | V245 | V246 |
| Normalized frequency of zeta L (Maxfield and Scheraga 1976) | M124 | V247 | V248 |
| Normalized frequency of the alpha region (Maxfield and Scheraga 1976) | M125 | V249 | V250 |
| Refractivity (McMeekin et al. 1964) Cited by Jones (1975) | M126 | V251 | V252 |
| Retention coefficient in HPLC pH7.4 (Meek 1980) | M127 | V253 | V254 |
| Retention coefficient in HPLC pH2.1 (Meek 1980) | M128 | V255 | V256 |
| Retention coefficient in NaH2PO4 (Meek and Rossetti 1981) | M129 | V257 | V258 |
| Average reduced distance for the C-alpha (Meirovitch et al. 1980) | M130 | V259 | V260 |
| Average reduced distance for a side chain (Meirovitch et al. 1980) | M131 | V261 | V262 |
| Normalized frequency of the alpha-helix (Nagano 1973) | M132 | V263 | V264 |
| Normalized frequency of the beta-structure (Nagano 1973) | M133 | V265 | V266 |
| Normalized frequency of the coil (Nagano 1973) | M134 | V267 | V268 |
| AA composition of total proteins (Nakashima et al. 1990) | M135 | V269 | V270 |
| AA composition of mt-proteins (Nakashima et al. 1990) | M136 | V271 | V272 |
| Normalized composition of mt-proteins (Nakashima et al. 1990) | M137 | V273 | V274 |
| Normalized composition from an animal (Nakashima et al. 1990) | M138 | V275 | V276 |
| AA composition of mt-proteins from fungi and plants (Nakashima et al. 1990) | M139 | V277 | V278 |
| Normalized composition from fungi and plants (Nakashima et al. 1990) | M140 | V279 | V280 |
| Normalized composition of membrane proteins (Nakashima et al. 1990) | M141 | V281 | V282 |
| Transmembrane regions of mt-proteins (Nakashima et al. 1990) | M142 | V283 | V284 |
| Ratio of average and computed composition (Nakashima et al. 1990) | M143 | V285 | V286 |
| AA composition of CYT of single-spanning proteins (Nakashima and Nishikawa 1992) | M144 | V287 | V288 |
| AA composition of CYT2 of single-spanning proteins (Nakashima and Nishikawa 1992) | M145 | V289 | V290 |
| AA composition of MEM of single-spanning proteins (Nakashima and Nishikawa 1992) | M146 | V291 | V292 |

| | | | |
|---|---|---|---|
| AA composition of MEM of multi-spanning proteins (Nakashima and Nishikawa 1992) | M147 | V293 | V294 |
| 14 A contact number (Nishikawa and Ooi 1986) | M148 | V295 | V296 |
| Transfer energy of organic solvent/water (Nozaki and Tanford 1971) | M149 | V297 | V298 |
| Average non-bonded energy per atom (Oobatake and Ooi 1977) | M150 | V299 | V300 |
| Short and medium range non-bonded energy per atom (Oobatake and Ooi 1977) | M151 | V301 | V302 |
| Average non-bonded energy per residue (Oobatake and Ooi 1977) | M152 | V303 | V304 |
| Short and medium range non-bonded energy per residue (Oobatake and Ooi 1977) | M153 | V305 | V306 |
| Optimized transfer energy parameter (Oobatake et al. 1985) | M154 | V307 | V308 |
| Optimized average non-bonded energy per atom (Oobatake et al. 1985) | M155 | V309 | V310 |
| Optimized side chain interaction parameter (Oobatake et al. 1985) | M156 | V311 | V312 |
| Normalized frequency of the alpha-helix from LG (Palau et al. 1981) | M157 | V313 | V314 |
| Normalized frequency of the alpha-helix from CF (Palau et al. 1981) | M158 | V315 | V316 |
| Normalized frequency of the beta-sheet from LG (Palau et al. 1981) | M159 | V317 | V318 |
| Normalized frequency of the beta-sheet from CF (Palau et al. 1981) | M160 | V319 | V320 |
| Normalized frequency of a turn from LG (Palau et al. 1981) | M161 | V321 | V322 |
| Normalized frequency of an alpha-helix in the all-alpha class (Palau et al. 1981) | M162 | V323 | V324 |
| Normalized frequency of an alpha-helix in the alpha+beta class (Palau et al. 1981) | M163 | V325 | V326 |
| Normalized frequency of a beta-sheet in the all-beta class (Palau et al. 1981) | M164 | V327 | V328 |
| Normalized frequency of a beta-sheet in the alpha beta class (Palau et al. 1981) | M165 | V329 | V330 |
| Normalized frequency of a beta-sheet in the alpha/beta class (Palau et al. 1981) | M166 | V331 | V332 |
| Normalized frequency of a turn in the all-alpha class (Palau et al. 1981) | M167 | V333 | V334 |
| Normalized frequency of a turn in the all-beta class (Palau et al. 1981) | M168 | V335 | V336 |
| Normalized frequency of a turn in the alpha/beta class (Palau et al. 1981) | M169 | V337 | V338 |
| HPLC parameter (Parker et al. 1986) | M170 | V339 | V340 |
| Partition coefficient (Pliska et al. 1981) | M171 | V341 | V342 |
| Average gain ratio in the surrounding hydrophobicity (Ponnuswamy et al. 1980) | M172 | V343 | V344 |
| Surrounding hydrophobicity in the alpha-helix (Ponnuswamy et al. 1980) | M173 | V345 | V346 |
| Surrounding hydrophobicity in the turn (Ponnuswamy et al. 1980) | M174 | V347 | V348 |
| Accessibility reduction ratio (Ponnuswamy et al. 1980) | M175 | V349 | V350 |
| Average number of surrounding residues (Ponnuswamy et al. 1980) | M176 | V351 | V352 |
| Hydrophobicity (Prabhakaran 1990) | M177 | V353 | V354 |
| Relative frequency in the alpha-helix (Prabhakaran 1990) | M178 | V355 | V356 |
| Relative frequency in the reverse-turn (Prabhakaran 1990) | M179 | V357 | V358 |
| Helix-coil equilibrium constant (Ptitsyn and Finkelstein 1983) | M180 | V359 | V360 |
| Beta-coil equilibrium constant (Ptitsyn and Finkelstein 1983) | M181 | V361 | V362 |
| Weights for an alpha-helix at the window position of -5 (Qian and Sejnowski 1988) | M182 | V363 | V364 |
| Weights for an alpha-helix at the window position of -3 (Qian and Sejnowski 1988) | M183 | V365 | V366 |
| Weights for an alpha-helix at the window position of -2 (Qian and Sejnowski 1988) | M184 | V367 | V368 |

| | | | |
|---|---|---|---|
| Weights for an alpha-helix at the window position of -1 (Qian and Sejnowski 1988) | M185 | V369 | V370 |
| Weights for an alpha-helix at the window position of 2 (Qian and Sejnowski 1988) | M186 | V371 | V372 |
| Weights for an alpha-helix at the window position of 3 (Qian and Sejnowski 1988) | M187 | V373 | V374 |
| Weights for an alpha-helix at the window position of 4 (Qian and Sejnowski 1988) | M188 | V375 | V376 |
| Weights for an alpha-helix at the window position of 5 (Qian and Sejnowski 1988) | M189 | V377 | V378 |
| Weights for a beta-sheet at the window position of -6 (Qian and Sejnowski 1988) | M190 | V379 | V380 |
| Weights for a beta-sheet at the window position of -5 (Qian and Sejnowski 1988) | M191 | V381 | V382 |
| Weights for a beta-sheet at the window position of -3 (Qian and Sejnowski 1988) | M192 | V383 | V384 |
| Weights for a beta-sheet at the window position of -2 (Qian and Sejnowski 1988) | M193 | V385 | V386 |
| Weights for a beta-sheet at the window position of -1 (Qian and Sejnowski 1988) | M194 | V387 | V388 |
| Weights for a beta-sheet at the window position of 0 (Qian and Sejnowski 1988) | M195 | V389 | V390 |
| Weights for a beta-sheet at the window position of 1 (Qian and Sejnowski 1988) | M196 | V391 | V392 |
| Weights for a beta-sheet at the window position of 2 (Qian and Sejnowski 1988) | M197 | V393 | V394 |
| Weights for a beta-sheet at the window position of 3 (Qian and Sejnowski 1988) | M198 | V395 | V396 |
| Weights for a beta-sheet at the window position of 4 (Qian and Sejnowski 1988) | M199 | V397 | V398 |
| Weights for a beta-sheet at the window position of 5 (Qian and Sejnowski 1988) | M200 | V399 | V400 |
| Weights for a coil at the window position of -6 (Qian and Sejnowski 1988) | M201 | V401 | V402 |
| Weights for a coil at the window position of -5 (Qian and Sejnowski 1988) | M202 | V403 | V404 |
| Weights for a coil at the window position of -4 (Qian and Sejnowski 1988) | M203 | V405 | V406 |
| Weights for a coil at the window position of -3 (Qian and Sejnowski 1988) | M204 | V407 | V408 |
| Weights for a coil at the window position of -2 (Qian and Sejnowski 1988) | M205 | V409 | V410 |
| Weights for a coil at the window position of -1 (Qian and Sejnowski 1988) | M206 | V411 | V412 |
| Weights for a coil at the window position of 0 (Qian and Sejnowski 1988) | M207 | V413 | V414 |
| Weights for a coil at the window position of 1 (Qian and Sejnowski 1988) | M208 | V415 | V416 |
| Weights for a coil at the window position of 2 (Qian and Sejnowski 1988) | M209 | V417 | V418 |
| Weights for a coil at the window position of 3 (Qian and Sejnowski 1988) | M210 | V419 | V420 |
| Weights for a coil at the window position of 4 (Qian and Sejnowski 1988) | M211 | V421 | V422 |
| Weights for a coil at the window position of 5 (Qian and Sejnowski 1988) | M212 | V423 | V424 |
| Weights for a coil at the window position of 6 (Qian and Sejnowski 1988) | M213 | V425 | V426 |
| Side chain orientational preference (Rackovsky and Scheraga 1977) | M214 | V427 | V428 |
| Average relative fractional occurrence in A0(i) (Rackovsky and Scheraga 1982) | M215 | V429 | V430 |
| Average relative fractional occurrence in AR(i) (Rackovsky and Scheraga 1982) | M216 | V431 | V432 |
| Average relative fractional occurrence in A0(i-1) (Rackovsky and Scheraga 1982) | M217 | V433 | V434 |
| Average relative fractional occurrence in AR(i-1) (Rackovsky and Scheraga 1982) | M218 | V435 | V436 |
| Average relative fractional occurrence in AL(i-1) (Rackovsky and Scheraga 1982) | M219 | V437 | V438 |
| Average relative fractional occurrence in ER(i-1) (Rackovsky and Scheraga 1982) | M220 | V439 | V440 |
| Value of theta(i) (Rackovsky and Scheraga 1982) | M221 | V441 | V442 |
| Transfer free energy from chx to wat (Radzicka and Wolfenden 1988) | M222 | V443 | V444 |

| | | | |
|---|---|---|---|
| Transfer free energy from vap to chx (Radzicka and Wolfenden 1988) | M223 | V445 | V446 |
| Transfer free energy from chx to oct (Radzicka and Wolfenden 1988) | M224 | V447 | V448 |
| Accessible surface area (Radzicka and Wolfenden 1988) | M225 | V449 | V450 |
| Energy transfer from out to in(95%buried) (Radzicka and Wolfenden 1988) | M226 | V451 | V452 |
| Relative preference value at N" (Richardson and Richardson 1988) | M227 | V453 | V454 |
| Relative preference value at N-cap (Richardson and Richardson 1988) | M228 | V455 | V456 |
| Relative preference value at N1 (Richardson and Richardson 1988) | M229 | V457 | V458 |
| Relative preference value at N2 (Richardson and Richardson 1988) | M230 | V459 | V460 |
| Relative preference value at N3 (Richardson and Richardson 1988) | M231 | V461 | V462 |
| Relative preference value at N5 (Richardson and Richardson 1988) | M232 | V463 | V464 |
| Relative preference value at Mid (Richardson and Richardson 1988) | M233 | V465 | V466 |
| Relative preference value at C5 (Richardson and Richardson 1988) | M234 | V467 | V468 |
| Relative preference value at C4 (Richardson and Richardson 1988) | M235 | V469 | V470 |
| Relative preference value at C1 (Richardson and Richardson 1988) | M236 | V471 | V472 |
| Relative preference value at C1 (Richardson and Richardson 1988) | M237 | V473 | V474 |
| Relative preference value at C11 (Richardson and Richardson 1988) | M238 | V475 | V476 |
| Information measure for an alpha-helix (Robson and Suzuki 1976) | M239 | V477 | V478 |
| Information measure for an N-terminal helix (Robson and Suzuki 1976) | M240 | V479 | V480 |
| Information measure for a middle helix (Robson and Suzuki 1976) | M241 | V481 | V482 |
| Information measure for a C-terminal helix (Robson and Suzuki 1976) | M242 | V483 | V484 |
| Information measure for an extended without H-bond (Robson and Suzuki 1976) | M243 | V485 | V486 |
| Information measure for an N-terminal turn (Robson and Suzuki 1976) | M244 | V487 | V488 |
| Information measure for a middle turn (Robson and Suzuki 1976) | M245 | V489 | V490 |
| Information measure for a C-terminal turn (Robson and Suzuki 1976) | M246 | V491 | V492 |
| Information measure for a coil (Robson and Suzuki 1976) | M247 | V493 | V494 |
| Information measure for a loop (Robson and Suzuki 1976) | M248 | V495 | V496 |
| Hydration free energy (Robson and Osguthorpe 1979) | M249 | V497 | V498 |
| Mean area buried on transfer (Rose et al. 1985) | M250 | V499 | V500 |
| Side chain hydropathy corrected for solvation (Roseman 1988) | M251 | V501 | V502 |
| Loss of Side chain hydropathy by helix formation (Roseman 1988) | M252 | V503 | V504 |
| Transfer free energy (Simon 1976) Cited by Charton and Charton (1982) | M253 | V505 | V506 |
| Principal component I (Sneath 1966) | M254 | V507 | V508 |
| Principal component II (Sneath 1966) | M255 | V509 | V510 |
| Principal component III (Sneath 1966) | M256 | V511 | V512 |
| Principal component IV (Sneath 1966) | M257 | V513 | V514 |
| Normalized frequency of an alpha-helix (Tanaka and Scheraga 1977) | M258 | V515 | V516 |
| Normalized frequency of an isolated helix (Tanaka and Scheraga 1977) | M259 | V517 | V518 |
| Normalized frequency of an extended structure (Tanaka and Scheraga 1977) | M260 | V519 | V520 |

| | | | |
|---|---|---|---|
| Normalized frequency of chain reversal R (Tanaka and Scheraga 1977) | M261 | V521 | V522 |
| Normalized frequency of chain reversal S (Tanaka and Scheraga 1977) | M262 | V523 | V524 |
| Normalized frequency of chain reversal D (Tanaka and Scheraga 1977) | M263 | V525 | V526 |
| Normalized frequency of a left-handed helix (Tanaka and Scheraga 1977) | M264 | V527 | V528 |
| Normalized frequency of zeta R (Tanaka and Scheraga 1977) | M265 | V529 | V530 |
| Normalized frequency of a coil (Tanaka and Scheraga 1977) | M266 | V531 | V532 |
| Relative population of conformational state A (Vasquez et al. 1983) | M267 | V533 | V534 |
| Relative population of conformational state C (Vasquez et al. 1983) | M268 | V535 | V536 |
| Relative population of conformational state E (Vasquez et al. 1983) | M269 | V537 | V538 |
| Electron-ion interaction potential (Veljkovic et al. 1985) | M270 | V539 | V540 |
| Transfer free energy to the lipophilic phase (von Heijne and Blomberg 1979) | M271 | V541 | V542 |
| Average interactions per side chain atom (Warme and Morgan 1978) | M272 | V543 | V544 |
| RF value in high salt chromatography (Weber and Lacey 1978) | M273 | V545 | V546 |
| Free energy change of epsilon(i) to epsilon(ex) (Wertz and Scheraga 1978) | M274 | V547 | V548 |
| Polar requirement (Woese 1973) | M275 | V549 | V550 |
| Hydration potential (Wolfenden et al. 1981) | M276 | V551 | V552 |
| Principal property value z1 (Wold et al. 1987) | M277 | V553 | V554 |
| Principal property value z2 (Wold et al. 1987) | M278 | V555 | V556 |
| Principal property value z3 (Wold et al. 1987) | M279 | V557 | V558 |
| Unfolding Gibbs energy in water pH7.0 (Yutani et al. 1987) | M280 | V559 | V560 |
| Unfolding Gibbs energy in water pH9.0 (Yutani et al. 1987) | M281 | V561 | V562 |
| Activation Gibbs energy of unfolding pH7.0 (Yutani et al. 1987) | M282 | V563 | V564 |
| Activation Gibbs energy of unfolding pH9.0 (Yutani et al. 1987) | M283 | V565 | V566 |
| Polarity (Zimmerman et al. 1968) | M284 | V567 | V568 |
| Isoelectric point (Zimmerman et al. 1968) | M285 | V569 | V570 |
| RF rank (Zimmerman et al. 1968) | M286 | V571 | V572 |
| Normalized positional residue frequency at helix termini N41(Aurora and Rose 1998) | M287 | V573 | V574 |
| Normalized positional residue frequency at helix termini N111 (Aurora and Rose 1998) | M288 | V575 | V576 |
| Normalized positional residue frequency at helix termini N11 (Aurora and Rose 1998) | M289 | V577 | V578 |
| Normalized positional residue frequency at helix termini N1(Aurora and Rose 1998) | M290 | V579 | V580 |
| Normalized positional residue frequency at helix termini N1 (Aurora and Rose 1998) | M291 | V581 | V582 |
| Normalized positional residue frequency at helix termini N4 (Aurora and Rose 1998) | M292 | V583 | V584 |
| Normalized positional residue frequency at helix termini N5 (Aurora and Rose 1998) | M293 | V585 | V586 |
| Normalized positional residue frequency at helix termini C5 (Aurora and Rose 1998) | M294 | V587 | V588 |
| Normalized positional residue frequency at helix termini C4 (Aurora and Rose 1998) | M295 | V589 | V590 |
| Normalized positional residue frequency at helix termini C2 (Aurora and Rose 1998) | M296 | V591 | V592 |
| Normalized positional residue frequency at helix termini C1 (Aurora and Rose 1998) | M297 | V593 | V594 |
| Normalized positional residue frequency at helix termini Cc (Aurora and Rose 1998) | M298 | V595 | V596 |

| | | | |
|---|---|---|---|
| Normalized positional residue frequency at helix termini C1 (Aurora and Rose 1998) | M299 | V597 | V598 |
| Normalized positional residue frequency at helix termini C11 (Aurora and Rose 1998) | M300 | V599 | V600 |
| Normalized positional residue frequency at helix termini C111 (Aurora and Rose 1998) | M301 | V601 | V602 |
| Normalized positional residue frequency at helix termini C41 (Aurora and Rose 1998) | M302 | V603 | V604 |
| Delta G values for the peptides extrapolated to 0 M urea (O1Neil and DeGrado 1990) 2 | M303 | V605 | V606 |
| Helix formation parameters (delta delta G) (O1Neil and DeGrado 1990) | M304 | V607 | V608 |
| Normalized flexibility parameters (B-values) average (Vihinen et al. 1994) | M305 | V609 | V610 |
| Normalized flexibility parameters (B-values) for each residue surrounded by one rigid neighbours (Vihinen et al. 1994) 2 | M306 | V611 | V612 |
| Free energy in the alpha-helical conformation (Munoz and Serrano 1994) | M307 | V613 | V614 |
| Free energy in the alpha-helical region (Munoz and Serrano 1994) 2 | M308 | V615 | V616 |
| Free energy in the beta-strand conformation (Munoz and Serrano 1994) | M309 | V617 | V618 |
| Free energy in the beta-strand region (Munoz and Serrano 1994) 2 | M310 | V619 | V620 |
| Free energies of transfer of AcWl-X-LL peptides from the bilayer interface to water (Wimley and White 1996) | M311 | V621 | V622 |
| Turn propensity scale for transmembrane helices (Monne et al. 1999) | M312 | V623 | V624 |
| Alpha-helix propensity of position 44 in T4 lysozyme (Blaber et al. 1993) | M313 | V625 | V626 |
| p-Values of mesophilic proteins based on the distributions of B values (Parthasarathy and Murthy 2000) | M314 | V627 | V628 |
| p-Values of thermophilic proteins based on the distributions of B values (Parthasarathy and Murthy 2000) | M315 | V629 | V630 |
| Distribution of amino acid residues in the 18 non-redundant families of thermophilic proteins (Kumar et al. 2000) 2 | M316 | V631 | V632 |
| Distribution of amino acid residues in the 18 non-redundant families of mesophilic proteins (Kumar et al. 2000) 2 | M317 | V633 | V634 |
| Distribution of amino acid residues in the alpha-helices in thermophilic proteins (Kumar et al. 2000) | M318 | V635 | V636 |
| Side-chain contribution to protein stability (kJ/mol) (Takano and Yutani 2001) | M319 | V637 | V638 |
| Hydropathy scale based on self-information values in the two-state model (5% accessibility) (Naderi-Manesh et al. 2001) 2 | M320 | V639 | V640 |
| Hydropathy scale based on self-information values in the two-state model (16% accessibility) (Naderi-Manesh et al. 2001) 2 | M321 | V641 | V642 |
| Hydropathy scale based on self-information values in the two-state model (20% accessibility) (Naderi-Manesh et al. 2001) 2 | M322 | V643 | V644 |
| Hydropathy scale based on self-information values in the two-state model (25% accessibility) (Naderi-Manesh et al. 2001) 2 | M323 | V645 | V646 |
| Hydropathy scale based on self-information values in the two-state model (50% accessibility) (Naderi-Manesh et al. 2001) 2 | M324 | V647 | V648 |
| Alpha-helix propensity derived from designed sequences (Koehl and Levitt 1999) | M325 | V649 | V650 |
| Beta-sheet propensity derived from designed sequences (Koehl and Levitt 1999) | M326 | V651 | V652 |
| Composition of amino acids in extracellular proteins (percent) (Cedano et al. 1997) | M327 | V653 | V654 |
| Composition of amino acids in anchored proteins (percent) (Cedano et al. 1997) 2 | M328 | V655 | V656 |
| Composition of amino acids in intracellular proteins (percent) (Cedano et al. 1997) | M329 | V657 | V658 |
| Composition of amino acids in nuclear proteins (percent) (Cedano et al. 1997) | M330 | V659 | V660 |
| Surface composition of amino acids in intracellular proteins of thermophiles (percent) (Fukuchi and Nishikawa 2001) 2 | M331 | V661 | V662 |
| Surface composition of amino acids in extracellular proteins of mesophiles (percent) (Fukuchi and Nishikawa 2001) 2 | M332 | V663 | V664 |
| Surface composition of amino acids in nuclear proteins (percent) (Fukuchi and Nishikawa 2001) | M333 | V665 | V666 |
| Interior composition of amino acids in intracellular proteins of thermophiles (percent) (Fukuchi and Nishikawa 2001) 2 | M334 | V667 | V668 |
| Interior composition of amino acids in nuclear proteins (percent) (Fukuchi and Nishikawa 2001) | M335 | V669 | V670 |
| Entire chain composition of amino acids in intracellular proteins of mesophiles (percent) (Fukuchi and Nishikawa 2001) 2 | M336 | V671 | V672 |

| | | | |
|---|---|---|---|
| Entire chain composition of amino acids in extracellular proteins of mesophiles (percent) (Fukuchi and Nishikawa 2001) 2 | M337 | V673 | V674 |
| Screening coefficients gamma local (Avbelj 2000) | M338 | V675 | V676 |
| Volumes including the crystallographic waters using the ProtOr (Tsai et al. 1999) | M339 | V677 | V678 |
| Electron–ion interaction potential values (Cosic 1994) | M340 | V679 | V680 |
| Hydrophobicity scales (Ponnuswamy 1993) | M341 | V681 | V682 |
| Hydrophobicity coefficient in RP-HPLC C8 with 0.1%TFA/MeCN/H2O (Wilce et al. 1995) | M342 | V683 | V684 |
| Hydrophobicity coefficient in RP-HPLC C4 with 0.1%TFA/MeCN/H2O (Wilce et al. 1995) | M343 | V685 | V686 |
| Hydrophilicity scale (Kuhn et al. 1995) | M344 | V687 | V688 |
| Retention coefficient at pH 2 (Guo et al. 1986) | M345 | V689 | V690 |
| Interactivity scale obtained by maximizing the mean of the correlation coefficient over single-domain globular proteins (Bastolla et al. 2005) 2 | M346 | V691 | V692 |
| Interactivity scale obtained by maximizing the mean of the correlation coefficient over pairs of sequences sharing the TIM barrel fold (Bastolla et al. 2005) 2 | M347 | V693 | V694 |
| Knowledge-based membrane-propensity scale from the 1D_Helix in MPtopo databases (Punta and Maritan 2003) 2 | M348 | V695 | V696 |
| Knowledge-based membrane-propensity scale from the 3D_Helix in MPtopo databases (Punta and Maritan 2003) 2 | M349 | V697 | V698 |
| Linker propensity from all datasets (George and Heringa 2003) | M350 | V699 | V700 |
| Linker propensity from the 1-linker dataset (George and Heringa 2003) | M351 | V701 | V702 |
| Linker propensity from the 2-linker dataset (George and Heringa 2003) | M352 | V703 | V704 |
| Linker propensity from the 3-linker dataset (George and Heringa 2003) | M353 | V705 | V706 |
| Linker propensity from the small dataset (linker length is less than six residues) (George and Heringa 2003) | M354 | V707 | V708 |
| Linker propensity from the long dataset (linker length is greater than 14 residues) (George and Heringa 2003) | M355 | V709 | V710 |
| Linker propensity from the helical (annotated by DSSP) dataset (George and Heringa 2003) | M356 | V711 | V712 |
| Linker propensity from the non-helical (annotated by DSSP) dataset (George and Heringa 2003) | M357 | V713 | V714 |
| The stability scale from the knowledge-based atom–atom potential (Zhou and Zhou 2004) | M358 | V715 | V716 |
| Buriability (Zhou and Zhou 2004) | M359 | V717 | V718 |
| Linker index (Bae et al. 2005) | M360 | V719 | V720 |
| Mean volumes of residues buried in protein interiors (Harpaz et al. 1994) | M361 | V721 | V722 |
| Average volumes of residues (Pontius et al. 1996) | M362 | V723 | V724 |
| Hydrophobicity index (Wolfenden et al. 1979) | M363 | V725 | V726 |
| Hydrophobicity-related index (Kidera et al. 1985) | M364 | V727 | V728 |
| Apparent partition energies calculated from the Wertz–Scheraga index (Guy 1985) | M365 | V729 | V730 |
| Apparent partition energies calculated from the Robson–Osguthorpe index (Guy 1985) | M366 | V731 | V732 |
| Hydrophobicity index at 3.0 pH (Cowan and Whittaker 1990) | M367 | V733 | V734 |
| Hydrophobicity scale from native protein structures (Casari and Sippl 1992) | M368 | V735 | V736 |
| PRIFT index (Cornette et al. 1987) | M369 | V737 | V738 |
| ALTFT index (Cornette et al. 1987) | M370 | V739 | V740 |
| TOTFT index (Cornette et al. 1987) | M371 | V741 | V742 |

**Table S5.** Comparison of the performance of the classifiers in seven major categories.

| Category | Classifier | ACC | MCC |
|---|---|---|---|
| bayes | BayesNet | 0.711 | 0.245 |
| | NaiveBayes | 0.719 | 0.259 |
| | NaiveBayesSimple | 0.719 | 0.259 |
| | NaiveBayesUpdateable | 0.719 | 0.259 |
| function | LibSVM | 0.781 | 0.436 |
| | Logistic | 0.734 | 0.291 |
| | RBFNetwork | 0.707 | 0.212 |
| | SMO | 0.706 | 0.203 |
| | Spegasos | 0.697 | 0.131 |
| | VotedPerceptron | 0.696 | -0.017 |
| lazy | IB1 | 0.758 | 0.428 |
| | IBK | 0.763 | 0.435 |
| | KStar | 0.817 | 0.547 |
| | LWL | 0.716 | 0.197 |
| meta | OrdinalClassClassifier | 0.775 | 0.423 |
| | RacedIncrementalLogitBoost | 0.697 | 0.047 |
| | RandomCommittee | 0.783 | 0.474 |
| | RandomSubspace | 0.780 | 0.434 |
| | RotationForest | 0.805 | 0.513 |
| | Stacking | 0.697 | 0.000 |
| | StackingC | 0.697 | 0.000 |
| | AdaBoostM1 | 0.697 | 0.000 |
| | AttributeSelectedClassifier | 0.755 | 0.357 |
| | Bagging | 0.769 | 0.415 |
| | ClassificationViaRegression | 0.568 | 0.054 |
| | ClassificationViaRegression | 0.747 | 0.339 |
| | CVParameterSelection | 0.697 | 0.000 |
| | Dagging | 0.726 | 0.241 |
| | Decorate | 0.792 | 0.479 |
| | END | 0.775 | 0.423 |
| | FilteredClassifier | 0.748 | 0.329 |
| | Grading | 0.697 | 0.000 |
| | LogitBoost | 0.722 | 0.230 |
| | MultiBoostAB | 0.697 | 0.000 |
| | MultiScheme | 0.697 | 0.000 |
| misc | HyperPipes | 0.697 | 0.029 |
| | VFI | 0.634 | 0.259 |
| rules | ConjunctiveRule | 0.697 | 0.000 |
| | DecisionTable | 0.742 | 0.321 |
| | DecisionTable.hashKey | 0.743 | 0.333 |
| | JRip | 0.744 | 0.345 |
| | OneR | 0.731 | 0.292 |
| | PART | 0.788 | 0.478 |
| | Ridor | 0.736 | 0.286 |
| | ZeroR | 0.697 | 0.000 |
| trees | ADTree | 0.700 | 0.136 |
| | BFtree | 0.773 | 0.441 |
| | DecisionStump | 0.697 | 0.000 |
| | FT | 0.747 | 0.367 |

| | | |
|---|---|---|
| J48 | 0.775 | 0.423 |
| J48greft | 0.775 | 0.423 |
| LADtree | 0.714 | 0.223 |
| LMT | 0.775 | 0.447 |
| NBTree | 0.783 | 0.456 |
| RandomForest | 0.784 | 0.474 |
| RandomTree | 0.774 | 0.459 |
| REPTree | 0.765 | 0.398 |
| SimpleCart | 0.775 | 0.449 |

**Table S6.** The performance of the ratios of positive and negative data. P: positive data; N: negative data.

| Ratio | *Sn* | *Sp* | *ACC* | *MCC* |
|---|---|---|---|---|
| **P:N = 1:2** | 0.625 | 0.895 | 0.805 | 0.547 |
| **P:N = 1:1.5** | 0.692 | 0.842 | 0.782 | 0.541 |
| **P:N = 1:1** | 0.758 | 0.749 | 0.754 | 0.507 |

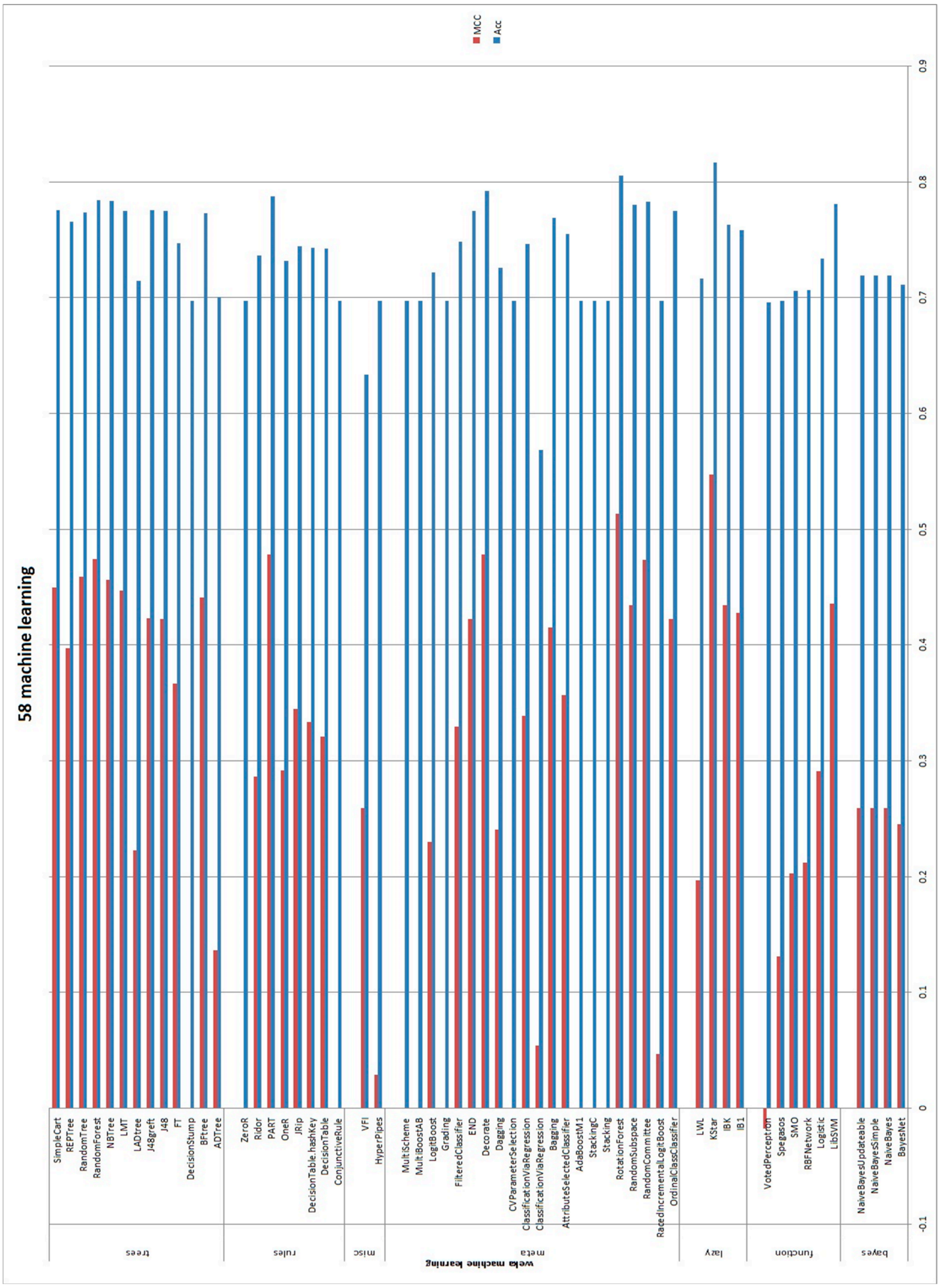


**Figure S1.** The evaluation and comparison of the classifiers' performance.

**Algorithm S1** Hill-Climbing algorithm used in KStable

```
Algorithm Hill-Climbing(fi)
 A = {a1, a2, a3, a4, a5} //basis feature
 B = {a1…a5, fi } //regular-mRMR feature fi
 F = {f1…fn} //features after regular-mRMR processing, sorted according to regular-mRMR
    fi = f1;
    do{
      if B.MCC() >= A.MCC() //Update if MCC improved
          A = B;
      else
          B = A;
      fn = move(F) //test next fn.
      B = B + {fn};
    }
    while F not isEnd()
    Return A
End Algorithm
```
